# Effects of a Short-Term Cycling Interval Session and Active Recovery on Non-Linear Dynamics of Cardiac Autonomic Activity in Endurance Trained Cyclists

**DOI:** 10.3390/jcm8020194

**Published:** 2019-02-06

**Authors:** Thomas Gronwald, Olaf Hoos, Kuno Hottenrott

**Affiliations:** 1 Department of Performance, Neuroscience, Therapy and Health, MSH Medical School Hamburg, University of Applied Sciences and Medical University, 20457 Hamburg, Germany; 2 Center for Sports and Physical Education, Julius Maximilians University of Wuerzburg, 97074 Würzburg, Germany; olaf.hoos@uni-wuerzburg.de; 3 Institute of Sports Science, Martin Luther University of Halle-Wittenberg, 06108 Halle (Saale), Germany; kuno.hottenrott@sport.uni-halle.de

**Keywords:** autonomic nervous system, HRV, Detrended Fluctuation Analysis, DFA, interval exercise, active recovery

## Abstract

Measurement of the non-linear dynamics of physiologic variability in a heart rate time series (HRV) provides new opportunities to monitor cardiac autonomic activity during exercise and recovery periods. Using the Detrended Fluctuation Analysis (DFA) technique to assess correlation properties, the present study examines the influence of exercise intensity and recovery on total variability and complexity in the non-linear dynamics of HRV. Sixteen well-trained cyclists performed interval sessions with active recovery periods. During exercise, heart rate (HR) and beat-to-beat (RR)-intervals were recorded continuously. HRV time domain measurements and fractal correlation properties were analyzed using the short-term scaling exponent alpha1 of DFA. Lactate (La) levels and the rate of perceived exertion (RPE) were also recorded at regular time intervals. HR, La, and RPE showed increased values during the interval blocks (*p* < 0.05). In contrast, meanRR and DFA-alpha1 showed decreased values during the interval blocks (*p* < 0.05). Also, DFA-alpha1 increased to the level in the warm-up periods during active recovery (*p* < 0.05) and remained unchanged until the end of active recovery (*p* = 1.000). The present data verify a decrease in the overall variability, as well as a reduction in the complexity of the RR-interval-fluctuations, owing to increased organismic demands. The acute increase in DFA-alpha1 following intensity-based training stimuli in active recovery may be interpreted as a systematic reorganization of the organism with increased correlation properties in cardiac autonomic activity in endurance trained cyclists.

## 1. Introduction

In recent years, analytics conducted with non-linear dynamics and chaos theory have been adapted to gain further insights into the complex cardiovascular regulation and exercise fatigue [1,2]. Thus, measures of the complexity of physiologic variability of heart rate time series, such as heart rate variability (HRV), may provide new opportunities to monitor cardiac autonomic activity during exercise. The present body of research suggests that cardiac dynamics is controlled by complex interactions between the sympathetic and parasympathetic branches of the autonomous nervous system on the sinus node and non-neural factors [3]. These two branches act competitively, resulting in a clear sympathetic activation and parasympathetic withdrawal during exercise [4]. Evaluation of absolute HRV values of time and frequency domain shows that exercise induces diminished variability even at low to moderate exercise intensities. Findings derived from such linear parameters have led to inconsistent results during different exercise intensities [4,5].

In healthy individuals, the HRV signal is mainly composed of quasi-periodic oscillations, but it also shows random fluctuations and so-called fractal structures [6]. Analysis of these structures has become a popular tool useful in the investigation of age and disease [7]. In this respect, analysis methods of non-linear dynamics in HRV do not describe the amplitude of the variability, but qualitative characteristics of the structure and dynamics of the signal. One widely applied approach to investigate scaling characteristics is the Detrended Fluctuation Analysis (DFA). This analysis provides a differentiated view of the random variability from the variability caused by physiological processes in the time series [1,6]. Thus, the DFA is a non-linear method to quantify the fractal scale and the degree of correlation within an HRV signal in the form of a dimensionless measurement. It should be noted that the short-term scaling exponent of DFA has already been applied for cardiovascular risk assessment as well as prognosis in clinical settings [8,9,10,11]. The current body of research in this field shows that regardless of the disease or age group investigated, values that differ from the normal value of approximately 1.0 for the short-term scaling exponent (decreasing or increasing) are associated with higher morbidity or worse prognosis. This indicates a loss of fractal dynamics towards random (disorganized randomness) or strongly correlated (periodicity) behavior [12]. This may be related to the maintenance of basic stability of the control systems between order (persistence) and disorder (change) in the context of homeodynamics [13,14,15]. This is one mechanism by which a complex biological network can avoid too much order, but also too much chaos, to remain close to a critical threshold under resting conditions. When physiologic systems lose their fractal complexity, they are less adaptable and less able to cope with varied stimuli such as exposure to different modes of exercise or changing environmental conditions [16].

As DFA provides robustness against artefacts and has a low dependence on heart rate [4,9], this method is suitable for analyzing the complexity of cardiovascular regulation in terms of the autonomic nervous system activity during various exercise modalities and intensities. Consequently, in addition to time- and frequency-domain measures of HRV, some studies have used DFA to analyze the time series during different exercise modes [1,17,18,19,20,21,22,23]. Previous studies that have investigated the isolated influence of different exercise modes on HRV have shown a decrease in variability and a loss of complexity with increasing exercise intensity in incremental exercise test settings [17,18,21]. In recent studies investigating incremental cycling tests until voluntary exhaustion and tests with different cycling cadences, it was shown that the short-term scaling exponent DFA-alpha1 was very sensitive to changes in exercise intensity and varied movement frequencies [21,22,23]. Furthermore, a stronger reduction in DFA-alpha1 was inversely correlated with a higher aerobic capacity in young athletes, indicating a possible relevant relation between exercise adaptation and nonlinear cardiac autonomic regulation [21]. In this context, nonlinear fluctuations of HRV during exercise may be seen as an outcome of the complex dynamic interplay of electro-physiological, hemodynamic and humoral variables, along with autonomic and central nervous system regulation [21]. Thus, the measurement of non-linear HRV during exercise and recovery might help to gain further insight into the complex heart-brain integration response as a part of a general stress related regulation capacity.

Further studies are necessary to analyze other modes of exercise and their influence on the short-term scaling exponent, as well as changing characteristics in active and passive recovery periods. In addition, further studies could provide new insights for application of non-linear HRV measures as a control parameter in training and therapy [22]. Performance of non-linear HRV analysis during and after high-intensity interval exercise suggests relevant information underlying physiological recovery that could be used to assess organismic demands or cardiovascular risk, such as exercise interventions in the context of therapy, health care, and fitness. A recent review of the markers of cardiac autonomic recovery after exercise supports the application of HRV parameters for the assessment of cardiac health [24]. In this context, Medeiros et al. [25] emphasized the application of non-linear methods of HRV analysis as a maturing and crucial methodology for the assessment of recovery periods. In addition, non-linear methods have previously demonstrated high reliability in identifying changes in post-exercise HRV during active recovery [26].

It was the aim of the present study to analyze the influence of short-term interval sessions combined with active recovery periods on standard time-domain measures and non-linear dynamics of HRV. The main objective was to determine whether active recovery is effective to prevent the loss of fractal organization in the short-term scaled characteristic of cardiac autonomic activity in endurance trained cyclists. 

## 2. Materials and Methods

### 2.1. Participants

Sixteen endurance trained male cyclists (age: 25.9 ± 3.8 years; height: 180.7 ± 6.1 cm; body mass: 77.4 ± 8.2 kg; body fat: 12.3% ± 3.4%; VO_2_ peak: 54.1 ± 6.1 mL/min/kg) were recruited from local sports clubs. We included non-smoking adults that performed cycling training sessions for at least 8 h per week over the past 6 months. A preliminary medical check-up following the S1 guidelines of the German Association for Sports Medicine and Prevention was performed to ensure that the participants have no cardiovascular, neurologic, pulmonary or orthopedic problems. The check-up also included an electrocardiogram (ECG) at rest and personal anamnesis. Following the explanation of risks and benefits associated with the study, the participants provided written informed consent. All procedures were in line with the declaration of Helsinki and the study protocol was approved by the local ethics committee.

### 2.2. Procedure

**Figure 1:**
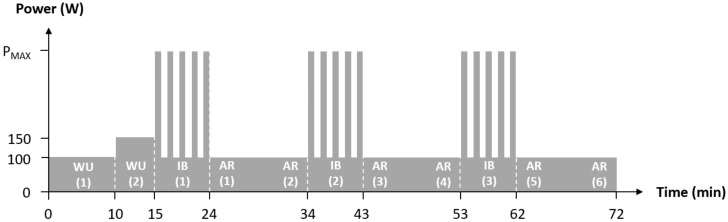
Flow chart of the interval session with active recovery periods; P_MAX_: Maximum power from the previous incremental cycling test until voluntary exertion; WU (1): Warm-Up at 100 W; WU (2): Warm-Up at 150 W; IB (1–3): Interval-Block 1–3; AR (1–6): Active-Recovery 1–6.

First, aerobic fitness in terms of oxygen uptake was assessed using spiroergometry (Metamax 3b, Fa. Cortex, Leipzig, Germany) during an incremental test until voluntary exhaustion (start: 100 W, increment: 20 W, length: 3 min) on a high-performance bicycle ergometer (E 2000 s, Fa. FES, Berlin, Germany). One week after the first laboratory visit, participants completed an interval session including 3 blocks of 5 intervals (60 s) at maximum power (P_MAX_) determined in the incremental test (see Figure 1) on the same bicycle ergometer and under the same conditions. Prior to the exercise bout, there was a warm-up at 100 W for 10 min followed by 150 W for 5 min. After each interval there was an active recovery at 100 W over a period of 60 s and after each interval block over a period of 10 min.

### 2.3. Measurements

During exercise, heart rate (HR) and beat-to-beat (RR)-intervals were recorded continuously using a HR-monitor with a time resolution of 1ms (Polar s810i, Fa. Polar Electro GmbH, Büttelborn, Germany; [27]). Collected raw data were transferred to a personal computer via an infrared interface. Subsequently, artefacts were detected with a semi-automatic approach. RR-intervals, which were distinguished by more than 30% difference from the previous interval, were determined as artefacts. Artefacts were replaced with the average calculated from previous and subsequent values, and data sets with more than 5% of artifacts were completely excluded from further processing. Only NN intervals (normal-to-normal intervals) were taken into account during the data analysis [28]. The NN intervals were stored as ASCII files for further data analysis.

Using Kubios HRV software (Version 2.1, Fa. Biosignal Analysis and Medical Imaging Group, Kuopio, Finland; [29]), HRV analysis was conducted on data collected from the last minute of the warm-up conditions and the interval blocks. In addition, during each active recovery period between the interval blocks the second and last minute was analyzed. Besides standard parameters obtained from time-domain analysis including the average of normal RR-interval length (meanRR in ms) and the root mean square of successive differences (RMSSD in ms), the scaling behavior was calculated using the non-linear short-term scaling exponent alpha1 of DFA (DFA-alpha1). DFA has been referred to as a modification of the root mean square analysis (RMS) that is also suitable for analyzing short and non-stationary time series data [9]. Briefly, the root mean square fluctuation of the integrated and detrended data is measured in observation windows of different sizes. The data were then plotted against the size of the window on a log-log scale. The scaling exponent represents the slope of the line, which relates (log) fluctuation to (log) window size [30]. In this study, we only computed the short term scaling exponent (window width: 4 ≤ *n* ≤ 16 beats) due to the relatively short recording times for each condition [18,20]. DFA-alpha1 values indicate time series fractal correlation properties, i.e., type of noise: Approx. 1.5 for strongly correlated Brownian noise and ≤0.5 for uncorrelated white noise with random signals. Approx. 1 signifies a mix of uncorrelated and maximally correlated signal components with 1/f noise (represents a balance between the complete unpredictability (randomness) of white noise and the predictability (strong correlations) of Brownian noise) [10]. Larger values of DFA-alpha1 represent a smoother time series and vice versa [6,9].

Additionally, blood lactate concentration (La) was assessed with Super GL ambulance (Fa. Dr. Mueller, Freital, Germany) from blood taken from an earlobe [31] and participants were asked to rate perceived exertion (RPE: 6–20; [32]). These measures were taken at the last minute of each test condition. All parameters were also assessed in resting state.

### 2.4. Statistical Analysis

Statistical analysis was performed with SPSS 23.0 (Fa. IBM Statistics, New York City, NY, USA) for Windows (Fa. Microsoft, Redmond, WA, USA). The Shapiro-Wilk test was applied to verify the Gaussian distribution of the data. The degree of variance homogeneity was verified by Levene test. Subsequently, a one-way ANOVA for repeated measurements was used to investigate the influence of the different conditions of the exercise bout (Interval-Block 1–3: IB (1), IB (2) and IB (3), Active-Recovery 1–6: AR (1), AC (2), AC (3), AR (4), AC (5) and AC (6)) on changes in HR, HRV parameters, La, and RPE. In case of significant main effects, post hoc tests (Bonferroni) were applied to compare differences between conditions. For the comparison in the warm-up periods, a *t*-test for paired samples was added. For all tests, statistical significance was accepted as *p* < 0.05. Eta^2^ was used to denote effect sizes.

## 3. Results

Participants achieved a maximum power output P_MAX_ of 338.3 ± 30.7 W during the incremental test. The individual P_MAX_ was taken over for the interval load. For all analyzed parameters a significant main effect of time could be determined (HR: *F*(8,15) = 360.551, *p* = 0.000, eta^2^ = 0.960; La: *F*(5,15) = 118.119, *p* = 0.000, eta^2^ = 0.887; RPE: *F*(5,15) = 77.052, *p* = 0.000, eta^2^ = 0.837; meanRR: *F*(8,15) = 219.597, *p* = 0.000, eta^2^ = 0.936; RMSSD: *F*(8,15) = 3.321, *p* = 0.037, eta^2^ = 0.181; DFA-alpha1: *F*(8,15) = 74.068, *p* = 0.000, eta^2^ = 0.832).

**Figure 2:**
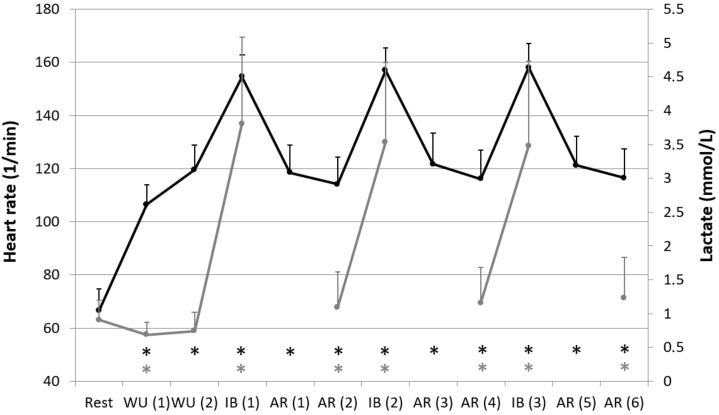
Heart rate (HR, black color) and blood lactate concentration (La, grey color) during resting state and all cycling conditions; WU (1): Warm-Up at 100 W; WU (2): Warm-Up at 150 W; IB (1–3): Interval-Block 1–3; AR (1–6): Active-Recovery 1–6; * significant compared to preceding measurement (*p* < 0.05).

**Figure 3:**
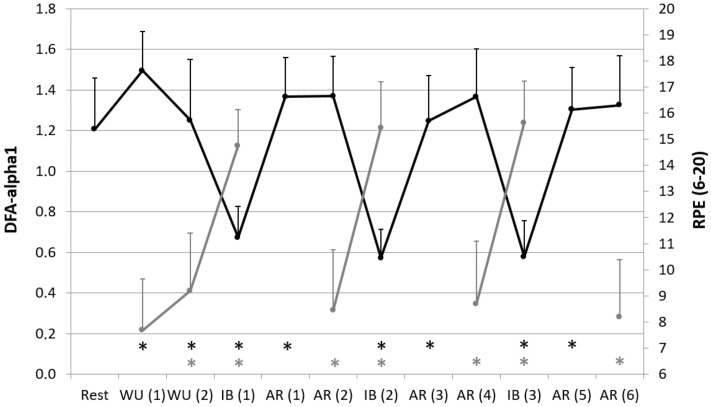
Short-term scaling exponent (DFA-alpha1, black color) and rate of perceived exertion (RPE, grey color) during resting state and all cycling conditions; WU (1): Warm-Up at 100 W; WU (2): Warm-Up at 150 W; IB (1–3): Interval-Block 1–3; AR (1–6): Active-Recovery 1–6; * significant compared to preceding measurement (*p* < 0.05).

In comparison of the test conditions, a clear influence of the different intensities could be determined. HR, La, and RPE showed significant increased values during the interval blocks (WU (2) vs. IB (1) HR: *p* = 0.000; La: *p* = 0.000; RPE: *p* = 0.000; AR (2) vs. IB (2) HR: *p* = 0.000; La: *p* = 0.000; RPE: *p* = 0.000; AR (4) vs. IB (3): HR: *p* = 0.000; La: *p* = 0.000; RPE: *p* = 0.000; Table 1, Figure 2 and Figure 3). In contrast, all HRV values except RMSSD showed significant decreased values during the interval blocks (WU (2) vs. IB (1) meanRR: *p* = 0.000; RMSSD: *p* = 0.041; DFA-alpha1: *p* = 0.000; AR (2) vs. IB (2) meanRR: *p* = 0.000; RMSSD: *p* = 1.000; DFA-alpha1: *p* = 0.000; AR (4) vs. IB (3): meanRR: *p* = 0.000; RMSSD: *p* = 1.000; DFA-alpha1: *p* = 0.000; Table 1 and Figure 3). We couldn’t find any differences in comparison of the different interval blocks. It was also shown that DFA-alpha1 increased significantly during the active recovery up to the level of the warm-up periods (IB (1) vs. AR (1): *p* = 0.000; IB (2) vs. AR (3): *p* = 0.000; IB (3) vs. AR (5): *p* = 0.000) and remained at nearly the same rate until the end of the active recovery period (AR (1) vs. AR (2): *p* = 1.000; AR (3) vs. AR (4): *p* = 1.000; AR (5) vs. AR (6): *p* = 1.000; Table 1 and Figure 3).

## 4. Discussion

The present data combining interval loads with active recovery periods show that the DFA-alpha1 values return to the level of the warm-up periods very quickly during active recovery and remain at nearly the same values until the end of the active recovery phase in endurance trained cyclists. Therefore, it can be ascertained that the strongly correlated characteristics of the short-term scaling exponent of DFA during passive recovery in sitting position [17] can’t be avoided by active recovery. In addition, the results of the present study indicate that the assessment of DFA-alpha1 allows a distinction between different exercise intensities in short-term cycling interval sessions. This observation is in line with findings of previous studies supporting a discrepancy of the DFA during incremental exercise load and during constant workload with varied cadences [17,18,19,21,22,23].

The effect of different exercise intensities is indicated by a change from a predominance of sympathetic activity during high-intensity exercise to a predominance of parasympathetic activity during low-intensity exercise or recovery (parasympathetic reactivation) [17,33,34,35,36], and may also be related to a counter regulation (overcompensation) of the organism to the prior interval load. In contrast, Millar et al. [37] stated that the acute increase in DFA-alpha1 following intensity-based training stimuli (supine passive recovery) may also be interpreted as an increase in sympathetic activity and/or a reduction in vagal activity. Millar et al. [37] found that after intensive exercise (multiple sprint interval sessions through performance in four Wingate Tests), higher DFA-alpha1 values occurred in the acute recovery period compared to lower pre-load period (single sprint interval session through performance in one Wingate Test). Goya-Esteban et al. [38] also examined DFA-alpha1 in the recovery phase after a test until voluntary exertion (significantly decreased values during exercise) and were able to determine values in the range of the pre-load immediately after exercise. The values increased slightly for up to five minutes after exercise. The organism responds with a highly correlated behavior and more order in the early recovery period. This behavior was also confirmed by Blasco-Lafarga et al. [39,40] after a high-intensity Judo-specific test-battery and Martínez-Navarro et al. [41] after an ultramarathon. Casties et al. [17] showed that DFA-alpha1 increases at the beginning of the recovery phase followed by renormalization within minutes to hours. The authors justified this behavior with a temporary degradation of the regulatory processes and concluded there is a systemic reorganization in the recovery phase with possibly increased correlation properties by the inclusion of a variety of regulative systems.

The present data also reflect the close relationship of DFA-alpha1 with other load parameters such as RPE. The values of DFA-alpha1 of approximately 0.6 and the RPE values of approximately 15 during the interval blocks were also reached in the previous incremental cycling test in a clearly submaximal workload with an RPE of approximately 15 (6th stage before voluntary exertion; [22]). A comparison with the data from the incremental test shows that the selected intensity and/or duration of the interval periods were too low for a high-intensity interval session. This was also shown by the recorded values of heart rate and blood lactate concentration in the present study. Nevertheless, it was an effective training stimulus with regard to a high exercise intensity to detect the change between the different conditions.

In the present study, a decrease in DFA-alpha1, which corresponded with increasing exercise intensity, verifies a demand-dependent change from strongly correlated to uncorrelated/stochastic or anti-correlated behavior of the RR-intervals and back to strongly correlated behavior in early recovery [10]. This is consistent with previous studies reporting an almost linear reduction of complexity and approximation of the RR data structure towards a merely random and anti-correlated signal for medium to high exercise intensity and increased organismic demands [1,17,18,19,21,22,23,42]. The loss of complexity of the RR time series during exercise is related to the breakdown of the equilibrium between the two branches of the autonomic nervous system due to the decreased parasympathetic activity and/or the increased sympathetic activity [4,43]. This particular change could be due to an organismic system withdrawal which aims to protect homeodynamic processes [17,19]. Besides these influences, the great loss of complexity might be a consequence of complementary neural mechanisms/circuits [44] to maintain locomotor-respiratory coupling during cycling in the context of coordination between heartbeat, breathing patterns and movement frequency (e.g., cadence) [17,21,23]. The coupling and influence of mechanical and neurological processes on the sinus node may arise from ribcage movements, blood pumping of the lower limbs and simultaneous changes in pressure waves [17]. From a general point of view this situation might be favorable to provide maximum aerobic power output for a short period of time, as a stronger reduction in DFA-alpha1 may be related to higher aerobic capacity [21], but a fast recovery seems mandatory as a medium to long-term loss of HRV complexity could be understood as detrimental for the stability of the organism, which is maintained by the Central Autonomous Network (CAN) integrating various internal and external stimuli [45]. In this context, the non-linear analysis of HRV could provide a new approach for the integrated and holistic evaluation of regulatory processes during exercise and recovery [46,47,48]. Overall, the application of DFA may provide new methods to analyze the relationship between exercise and altered cardiac autonomic activity or control. This could be useful in response to the concern over increased variability and weak reproducibility of frequency-domain HRV measures due to low total spectral power during or immediately after exercise [37,49,50].

## 5. Limitations

This study tested the applicability of the short-term scaling exponent DFA-alpha1 during a short-term interval session to evaluate the complexity of physiological changes during both interval load and active recovery periods. Unfortunately, it was not possible to evaluate HRV in the first minute of the active recovery periods due to the recording of other measures such as blood lactate concentration. Future studies should take a closer look at how DFA-alpha1 responds shortly after high exercise load and during the recovery processes, especially with time-variant measures. Future studies should also investigate the influence of respiration and other confounding factors [51] on DFA-alpha1 under medium to high exercise intensities. In order to minimize the environment’s external influence and to enable coupling processes, respiration was not prescribed and controlled. Allowing the need of spontaneous breathing might be a limitation, but it is necessary if we aim to analyze individual responses during exercise [21]. Perakakis et al. [52] and Weippert et al. [53] were able to demonstrate a negligible influence of respiration under resting conditions. Nevertheless, Weippert et al. [53] could not prove a significant influence of respiration at very low exercise intensity. However, the authors noted that respiratory activity must be considered as a potential contributor at rest and during light dynamic exercise.

In addition, we are also aware that human physiology and cardiovascular regulation during exercise is too complex and too dependent on certain conditions and assumptions to be broken down into a single key measure. Nevertheless, non-linear analysis of HRV with DFA enables a suitable approach with a differentiated and qualitative view of exercise physiology and it may be useful in combination with other applicable internal and external load measures for diagnostics and training control.

## 6. Conclusions

The present data show that active recovery periods do not prevent the loss of fractal organization of cardiac autonomic activity in endurance trained cyclists after short-term interval sessions. DFA-alpha1 values return to the level of the warm-up periods during acute active recovery periods. In addition, the study shows a decrease in DFA-alpha1 which went along with an increasing exercise intensity. This observation is in accordance with findings of previous studies during tests with incremental exercise intensity and constant workload with different cadences. This verifies a demand-dependent change from strongly correlated to uncorrelated/stochastic or anti-correlated behavior of the RR-intervals. Therefore, such non-linear parameters of HRV are suitable to distinguish between different organismic demands and may prove helpful to monitor different recovery states. Additionally, this approach provides a more systemic view of cardiovascular regulation in the context of complex models of exercise and active recovery. The available data suggest that DFA-alpha1 could be used as an adequate and easy-to-access load measure for training (therapy) and recovery. From a methodical and practical perspective of training and exercise science, it would be interesting to further investigate the influence of structured training and therapy interventions and different performance levels on the described processes of autonomic regulation. Those findings might lead to a broader understanding of the underlying mechanisms which, in turn, could open new possibilities to evaluate and enhance the state of health, disease or performance level.

## Figures and Tables

**Table 1:** Heart rate, lactate and HRV measures (mean ± standard deviation) during resting state and all cycling conditions; WU (1): Warm-Up at 100 W; WU (2): Warm-Up at 150 W; IB (1–3): Interval-Block 1–3; AR (1–6): Active-Recovery 1–6.

	Rest	WU (1)	WU (2)	IB (1)	AR (1)	AR (2)	IB (2)	AR (3)	AR (4)	IB (3)	AR (5)	AR (6)
HR (1/min)	66.5 ± 8.2	106.4 * ± 7.4	119.6 * ± 9.4	154.5 * ± 8.4	118.4 * ± 10.3	114.1 * ± 10.2	157.0 * ± 8.4	121.6 * ± 11.6	116.1 * ± 10.8	157.9 * ± 9.1	121.3 * ± 11.0	116.4 * ± 11.0
La (mmol/L)	0.91 ± 0.29	0.69 * ± 0.19	0.74 ± 0.28	3.81 * ± 1.28	-	1.09 * ± 0.53	3.54 * ± 1.18	-	1.16 * ± 0.52	3.48 * ± 1.25	-	1.23 * ± 0.60
RPE (6–20)	-	7.7 ± 2.0	9.2 * ± 2.2	14.8 * ± 1.4	-	8.4 * ± 2.3	15.4 * ± 1.8	-	8.7 * ± 2.4	15.6 * ± 1.6	-	8.2 * ± 2.2
meanRR (ms)	923 ± 108	566 * ± 39	505 * ± 38	390 * ± 22	510 * ± 43	530 * ± 46	384 * ± 22	498 * ± 47	521 * ± 46	381 * ± 23	499 * ± 43	519 * ± 46
RMSSD (ms)	56.3 ± 31.1	3.2 * ± 0.6	2.9 ± 0.7	2.5 * ± 0.5	3.1 * ± 0.4	2.8 ± 0.6	2.6 ± 0.5	3.0 ± 0.6	2.8 ± 0.6	2.6 ± 0.6	3.0 ± 0.6	2.8 ± 0.8
DFA-alpha1	1.21 ± 0.25	1.49 * ± 0.19	1.25 * ± 0.30	0.67 * ± 0.15	1.37 * ± 0.19	1.37 ± 0.20	0.57 * ± 0.14	1.25 * ± 0.22	1.37 ± 0.23	0.58 * ± 0.18	1.30 * ± 0.21	1.32 ± 0.24

HR: heart rate, La: blood lactate concentration, RPE: rate of perceived exertion, meanRR: average of normal RR intervals, RMSSD: root mean square of successive differences, DFA-alpha1: short-term scaling exponent of detrended fluctuation analysis; * significant compared to preceding measurement (*p* < 0.05)
